# Age-related Curves of AMH Using the Gen II, the picoAMH, and the Elecsys Assays in Women With Polycystic Ovary Syndrome

**DOI:** 10.1210/clinem/dgae153

**Published:** 2024-03-15

**Authors:** Federica Barbagallo, Kim van der Ham, Sten P Willemsen, Yvonne V Louwers, Joop S Laven

**Affiliations:** Division of Reproductive Endocrinology and Infertility, Department of Obstetrics and Gynecology, Erasmus University Medical Center, 3015 CN Rotterdam, The Netherlands; Department of Clinical and Experimental Medicine, University of Catania, 95123 Catania, Italy; Division of Reproductive Endocrinology and Infertility, Department of Obstetrics and Gynecology, Erasmus University Medical Center, 3015 CN Rotterdam, The Netherlands; Department of Biostatistics, Erasmus MC, University Medical Center, 3015 CN Rotterdam, The Netherlands; Division of Reproductive Endocrinology and Infertility, Department of Obstetrics and Gynecology, Erasmus University Medical Center, 3015 CN Rotterdam, The Netherlands; Division of Reproductive Endocrinology and Infertility, Department of Obstetrics and Gynecology, Erasmus University Medical Center, 3015 CN Rotterdam, The Netherlands

**Keywords:** polycystic ovary syndrome (PCOS), anti-müllerian hormone (AMH), age, nomograms, age-specific percentiles

## Abstract

**Context:**

Several challenges still exist to adopt the anti-müllerian hormone (AMH) as a marker of polycystic ovary morphology, as included in the recently updated international guideline. Although different evaluations of age- and assay-specific reference ranges have been published in the past few years, these studies have mainly been conducted in normo-ovulatory or infertile women.

**Objective:**

To develop an age-specific percentile distribution of AMH in patients with polycystic ovary syndrome (PCOS) measured by 3 different assays.

**Design:**

Retrospective cross-sectional study.

**Patients:**

A total of 2725 women aged 20 to 40 years with PCOS diagnosis were included.

**Interventions:**

Serum AMH measurement by the Gen II (Beckman Coulter), the picoAMH (Ansh Labs), and the Elecsys (Roche) assays.

**Main outcome measures:**

Age-specific percentile curves for all the assays and correlations between AMH, clinical, hormonal, and ultrasound characteristics.

**Results:**

Age-related nomograms for the 5th, 10th, 25th, 50th, 75th, 90th, and 95th percentiles of AMH were calculated using the Lambda-Mu-Sigma method for all the assays. AMH levels were significantly different between PCOS phenotypes. AMH levels were positively correlated to LH, LH/FSH ratio, testosterone, androstenedione, free androgen index, mean follicular number, and mean ovarian volume.

**Conclusion:**

To our knowledge, this is the first study reporting age-specific percentile nomograms of serum AMH levels measured by the Gen II, the picoAMH, and the Elecsys assays in a large population of women with PCOS. These findings may help to interpret AMH levels in patients with PCOS and facilitate the use of AMH as a diagnostic tool across age ranges.

Polycystic ovary syndrome (PCOS) is the most common endocrine disorder affecting women of reproductive age, with a reported prevalence of 8% to 13% (1, 2). It is a heterogeneous condition involving reproductive, endocrine, metabolic, and psychosocial symptoms, which varies across a woman's lifespan (2). According to the Rotterdam criteria, at least 2 of the following 3 criteria are needed to diagnose PCOS: (1) menstrual irregularity and/or ovulatory dysfunction (OD); (2) clinical and/or biochemical hyperandrogenism; and (3) polycystic ovary morphology (PCOM) (1). PCOM has been strongly correlated with the level of anti-müllerian hormone (AMH), which has been proposed as a marker of PCOM (3).

AMH is a glycoprotein hormone belonging to the TGF-β family. It is secreted by granulosa cells of preantral and small antral follicles (4). In women, AMH contributes to the regulation of folliculogenesis through different mechanisms. First, it inhibits the initial recruitment of primary follicles from the resting pool of primordial follicles. AMH also inhibits the sensitivity of antral follicles to FSH during cyclic recruitment (5). Furthermore, AMH reduces aromatase activity and the conversion of testosterone produced by the ovarian theca cells to 17β-estradiol, which in turn inhibits AMH (6). The ovarian granulosa cells start secreting AMH around the 36th week of gestation. AMH levels steadily increase to peak and plateau around age 25 years. Afterwards, its serum levels begin to decline until menopause, when AMH production ceases (7).

Serum AMH levels are reported to be significantly higher in women with PCOS compared with normal ovulatory women (8-10). However, several challenges still exist to adopt AMH as a diagnostic marker of PCOM in adults. These include the high variability of the assays used for the AMH measurement and the lack of international standard for AMH measurement (11). Indeed, during the years, several assays have been developed to measure AMH; some of them are currently used in clinical practice. They are directed against different specific AMH regions and, in turn, the total AMH concentration measured in circulation depends on the presence of specific AMH isoforms. In total, there are theoretically at least 7 potential AMH isoforms that could be present in blood (12, 13).

Different evaluations of age- and assay-specific reference ranges have been published in the past few years with significantly different results (14-17). However, these studies are difficult to compare because the inclusion criteria are different for each study. In addition, these studies have mainly been conducted in normo-ovulatory or infertile women with normal or low serum AMH levels, and little is known about the higher range of AMH levels, especially in women with PCOS.

Therefore, this study aimed to establish age-specific AMH percentile values in a large population of women with PCOS using the following assays for its measurement: (1) original Gen II AMH ELISA (Beckman Coulter, Inc., Webster, TX, USA); (2) picoAMH assay (Ansh Labs, Webster, Texas, USA); and (3) Elecsys AMH Plus assay (Roche Diagnostics International Ltd, Rothkreuz, Switzerland). We also evaluated the correlation between AMH with clinical, endocrine, and ultrasound features in our cohort of PCOS women.

## Materials and Methods

### Cohorts

In this retrospective single-center cross-sectional study, 2725 women who consulted the Reproductive Endocrinology and Infertility Clinic of the Erasmus University Medical Center, Rotterdam, The Netherlands, were included. The Medical Ethical Review Board of the Erasmus University Medical Center Rotterdam approved retrospective studies within this patient population, which includes women with ovulatory dysfunction (MEC-2020-0534). The study was conducted in accordance with the local legislation and institutional requirements. Written informed consent for participation was not required from the participants or the participants’ legal guardians/next of kin in accordance with national legislation and institutional requirements.

During the visit, extensive patient history, general health, family history, and previous and current use of medication were recorded. Physical examination included body weight, and height measurement and the body mass index (BMI) were calculated. PCOM was defined as 12 or more follicles in 1 or both ovaries (2-9 mm in diameter) and/or increased ovarian volume (>10 cm^3^) using an ultrasound probe of <8 MHz.

The blood samples were collected on the day of clinical examination and processed within 2 hours after withdrawal. Before the assessment of AMH by the PicoAMH and Elecsys assays, serum samples were stored at −20 °C. Endocrine evaluation included serum levels of LH, FSH, testosterone, androstenedione, SHBG, and AMH. PCOS was diagnosed according to Rotterdam Criteria, described previously (1). Hyperandrogenism was assessed and classified as either biochemical or clinical hyperandrogenism. Biochemical hyperandrogenism was defined as having a free androgen index (FAI) >2.9. Clinical hyperandrogenism was defined as a modified Ferriman-Gallwey score >5 (18).

Women who underwent the aforementioned standardized screening and were diagnosed with PCOS were classified into 1 of the 4 potential PCOS phenotypes: (A) OD + hyperandrogenism + PCOM; (B) OD + hyperandrogenism; (C) hyperandrogenism + PCOM; or (D) OD + PCOM.

The following exclusion criteria were established: severe mental illness, ongoing pregnancy, fertility treatment during the study period, contraceptive hormonal therapies currently or in the past 3 months, and current treatment for malignancy.

After the identification of overall cohort, we restricted our analysis to only women between age 20 and 40 years to avoid the influence of estimation of too young or too older women.

### Hormonal Measurements

Serum AMH levels were measured using the following AMH assays: (1) Gen II AMH ELISA (catalog #79765, RRID: AB_2800500) (Beckman Coulter, Inc); (2) picoAMH assay (catalog #AL-124, RRID: AB_2783675) (Ansh Labs); and (3) Elecsys AMH plus assay (catalog #06331976, RRID: AB_2895131) (Roche Diagnostics International Ltd). Measurements with Beckman Coulter assays were performed at the diagnostic endocrine laboratory of Erasmus University Medical Center, Rotterdam. Measurements with the picoAMH and Elecsys AMH plus assays were performed at Ansh Labs and at a diagnostic laboratory in Rothkreuz, Switzerland, respectively. The intra- and inter-assay variability was below 5%. Both the Gen II AMH and the picoAMH are manual assays, whereas the Elecsys AMH plus assay is an automated assay. Both the picoAMH and the Elecsys assays have a capture antibody recognizing the C-terminal region, whereas the detector antibody recognizes the AMH_M_ region. The Gen II AMH assay uses a capture antibody recognizing an epitope in the AMH_N,229_ region and the detector antibody recognizing an epitope in the C-terminal region (19). AMH levels of Gen II, picoAMH, and Elecsys AMH assays are presented in ng/mL.

Serum levels of LH (Siemens catalog #L2KLH2, RRID:AB_2756388) and FSH (Siemens catalog #L2KFS2, RRID:AB_2756389) were measured with the use of immunoluminometric assays. Intra- and inter-assay coefficients of variation were, respectively, <3% and <6.2% for FSH, <3.5%, and <6.4% for LH. Levels of serum androstenedione, testosterone, were measured with liquid chromatography-tandem mass spectrometry. Intra- and inter-assay coefficients of variation were, respectively, <3% and <5% for testosterone and <8 and <11% for androstenedione. The FAI was calculated as (testosterone [nmol/L]/SHBG [nmol/L] × 100). The SHBG (Siemens catalog #L2KSH2, RRID:AB_2819251) was determined with the Immulite 2000 analyzer (Siemens Healthcare, Tarrytown, NY, USA) with intra- and inter-assay coefficient variations of less than 4% and less than 5%, respectively.

### Statistical Methods

Normality was assessed using the Kolmogorov-Smirnov test. Nonparametric tests were used when the variables were not normally distributed. Age-specific percentile curves were constructed for the Gen II, the picoAMH, and the Elecsys AMH assays using the Lambda-Mu-Sigma (LMS) method (20). This is done by assuming that the data have a normal distribution after a Box-Cox transformation. This is controlled by 3 parameters: a parameter L that controls the skewness, a parameter M that controls the location, and a parameter S for the scale. All 3 parameters (L, M, and S) are now modeled as a smooth function of age using splines. For each age, 7 percentiles (5th, 10th, 25th, 50th, 75th, 90th, and 95th) were tabulated. The relationship between serum AMH levels measured with each of 2 assays, clinical, hormonal, and ultrasound characteristics were quantified by Spearman correlation. Statistical analysis was performed using the software SPSS 28 for Windows (SPSS Inc., Chicago, IL, USA), and for the age percentiles curve Rstudio, version 3.6.1 was used. A *P* value lower than .05 was considered statistically significant.

## Results

### Cohorts’ Characteristics

In total, 2725 women with PCOS, aged between 20 and 40 years, were included in the study. The assessment of AMH was performed with both the picoAMH and the Elecsys AMH plus assay in 1756 women, with a median age of 28.6 (interquartile range, 25.4-31.6) years. The measurement of AMH was performed with the Gen II (Beckman Coulter) assay in 1702 patients with a median age of 29.3 (interquartile range, 26.1-32.6). Between these 2 cohorts, there is an overlap of 733 patients in whom AMH was measured with all 3 assays. Clinical, endocrine, and ultrasound characteristics of the patient cohorts are reported in Table 1.

**Table 1.** Clinical, endocrine, and ultrasound parameters (median and interquartile range) in cohorts enrolled

	Gen II Assay n = 1702	PicoAMH and Elecsys assay n = 1756
Clinical parameters		
Age (y)	29.3 (26.1-32.6)	28.6 (25.4-31.6)
BMI (kg/m^2^)	26.6 (22.2-31.6)	25.2 (21.8-30.4)
Age of menarche (y)	13 (12-14)	13 (12-14)
Endocrine parameters		
LH (IU/L)	7.5 (4.8-11.7)	8 (4.9-12.4)
FSH (IU/L)	5.5 (4.2-6.9)	5.8 (4.3-7.1)
LH/FSH	1.5 (1-2.2)	1.5 (0.9-2.2)
Testosterone (nmol/L)	1.3 (1-1.8)	1.5 (1.1-2.2)
FAI	3.3 (2-5.4)	4 (2.3-6.4)
Androstenedione (nmol/L)	6.1 (4.4-8.7)	8.9 (6-12.4)
SHBG (nmol/L)	41.4 (27.3-59.9)	41.1 (27.6-59.6)
Ultrasound parameters		
Mean number of follicles (both ovaries)	19.5 (14.5-28)	20 (14.5-28)
Ovarian volume (mL) (per ovary)	9.3 (6.9-12.5)	8.8 (6.6-11.9)

Abbreviations: BMI, body mass index; FAI, free androgen index.

### AMH Distribution in the Patients With PCOS

**Figure 1.**
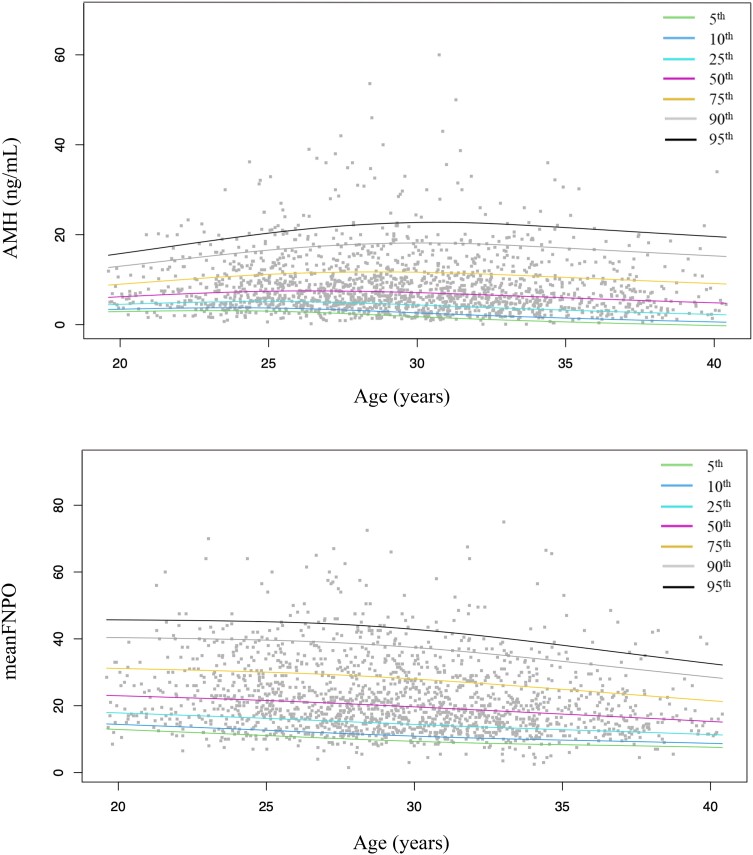
Cross-sectional age-specifics percentiles of anti-müllerian hormone and mean follicular number in patients with PCOS for the gen II (Beckman Coulter) assay.

The 5th, 10th, 25th, 50th, 75th, 90th, and 95th percentiles were calculated according to a population/based approach per age (years) for the Gen II (Table 2 and Fig. 1), the picoAMH (Table 3 and Supplementary Fig. S1 (23)), and the Elecsys (Table 4 and Supplementary Fig. S2 (23)) assays. According to the specific AMH cutoff previously reported in literature to diagnose PCOM for each assay, AMH values are shown in bold if greater than the proposed cutoff in Tables 2-4. In detail, we used the cutoffs of 5 and 5.5 ng/mL proposed by Lie Fong et al (21) for the Gen II assay, the cutoff of 6.16 ng/mL found by Bell et al for the picoAMH assay (22) and, last, the cutoff of 3.735 ng/mL validated by Zhang et al for the Elecsys assay (24). Considering these cutoffs, respectively, 33.9% (567/1673), 28.9% (478/1654), and 22.7% (375/1654) of women with PCOM showed AMH levels lower than previously proposed cutoffs for the Gen II, picoAMH, and Elecsys assays.

**Table 2.** Age-specific percentiles of anti-müllerian hormone in patients with PCOS for the Gen II (Beckman Coulter) assay

Age (y)	N	5th percentile	10th percentile	25th percentile	50th percentile	75th percentile	90th percentile	95th percentile
20	22	1.67	2.50	4.41	**6**.**27**	**8**.**69**	**11**.**83**	**19**.**23**
21	25	1.86	2.72	4.47	**6**.**69**	**9**.**84**	**13**.**53**	**19**.**52**
22	40	2.03	2.92	4.53	**7**.**08**	**10**.**89**	**15**.**10**	**19**.**84**
23	62	2.17	3.09	4.59	**7**.**39**	**11**.**72**	**16**.**43**	**20**.**24**
24	91	2.26	3.21	4.65	**7**.**61**	**12**.**28**	**17**.**45**	**20**.**73**
25	125	2.29	3.27	4.69	**7**.**71**	**12**.**47**	**18**.**04**	**21**.**36**
26	105	2.27	3.26	4.73	**7**.**65**	**12**.**23**	**18**.**14**	**22**.**13**
27	124	2.18	3.17	4.74	**7**.**50**	**11**.**73**	**17**.**91**	**22**.**80**
28	142	2.07	2.99	4.69	**7**.**29**	**11**.**30**	**17**.**69**	**22**.**97**
29	150	1.96	2.73	4.57	**7**.**12**	**11**.**20**	**17**.**77**	**22**.**33**
30	127	1.87	2.46	4.40	**6**.**97**	**11**.**29**	**17**.**96**	**21**.**28**
31	130	1.80	2.28	4.21	**6**.**79**	**11**.**28**	**17**.**96**	**20**.**41**
32	126	1.75	2.21	4.05	**6**.**58**	**11**.**05**	**17**.**66**	**19**.**97**
33	111	1.72	2.20	3.91	**6**.**34**	**10**.**70**	**17**.**19**	**19**.**78**
34	97	1.68	2.20	3.77	**6**.**11**	**10**.**29**	**16**.**70**	**19**.**64**
35	57	1.60	2.17	3.50	**5**.**67**	**9**.**53**	**15**.**82**	**19**.**25**
36	49	1.61	2.17	3.49	**5**.**62**	**9**.**45**	**15**.**61**	**19**.**24**
37	57	1.55	2.15	3.36	**5**.**47**	**9**.**15**	**15**.**41**	**19**.**00**
38	32	1.50	2.11	3.23	**5**.**27**	**8**.**78**	**15**.**02**	**18**.**74**
39	15	1.45	2.08	3.10	**5**.**07**	**8**.**42**	**14**.**65**	**18**.**47**
40	15	1.40	2.04	2.96	4.87	**8**.**06**	**14**.**28**	**18**.**19**

Abbreviations: AMH, anti-müllerian hormone; PCOS, polycystic ovary syndrome.

AMH values above the proposed cutoff in the study by Lie Fong et al, 2017 (21) are reported in bold.

**Table 3.** Age-specific percentiles of anti-müllerian hormone in patients with PCOS for the picoAMH (Ansh Labs) assay

Age (y)	N	5th percentile	10th percentile	25th percentile	50th percentile	75th percentile	90th percentile	95th percentile
20	19	2.44	2.83	5.66	**8**.**53**	**10**.**63**	**15**.**79**	**16**.**93**
21	48	2.28	2.86	5.62	**8**.**87**	**12**.**26**	**17**.**47**	**19**.**52**
22	68	2.16	2.93	5.60	**9**.**18**	**13**.**70**	**18**.**95**	**21**.**85**
23	93	2.14	3.08	5.63	**9**.**43**	**14**.**75**	**20**.**02**	**23**.**63**
24	97	2.25	3.36	5.74	**9**.**56**	**15**.**21**	**20**.**46**	**24**.**59**
25	130	2.55	3.82	5.94	**9**.**56**	**14**.**89**	**20**.**07**	**24**.**46**
26	142	2.95	4.35	**6**.**20**	**9**.**42**	**13**.**92**	**19**.**02**	**23**.**38**
27	122	3.18	4.62	**6**.**32**	**9**.**19**	**13**.**05**	**18**.**17**	**22**.**29**
28	146	2.97	4.32	6.15	**8**.**95**	**13**.**01**	**18**.**39**	**22**.**10**
29	169	2.49	3.64	5.78	**8**.**73**	**13**.**60**	**19**.**31**	**22**.**57**
30	132	2.21	3.19	5.51	**8**.**58**	**13**.**97**	**19**.**75**	**22**.**67**
31	126	2.32	3.18	5.46	**8**.**50**	**13**.**74**	**19**.**20**	**21**.**98**
32	114	2.58	3.37	5.49	**8**.**41**	**13**.**17**	**18**.**11**	**20**.**94**
33	98	2.77	3.50	5.44	**8**.**24**	**12**.**53**	**16**.**93**	**20**.**04**
34	73	2.77	3.47	5.26	**7**.**98**	**11**.**97**	**15**.**91**	**19**.**48**
35	51	2.62	3.29	4.96	**7**.**62**	**11**.**48**	**15**.**02**	**19**.**22**
36	41	2.32	2.98	4.56	**7**.**18**	**11**.**04**	**14**.**24**	**19**.**23**
37	34	1.91	2.57	4.07	**6**.**68**	**10**.**65**	**13**.**56**	**19**.**46**
38	21	1.42	2.07	3.51	6.13	**10**.**30**	**12**.**95**	**19**.**85**
39	17	0.86	1.51	2.90	5.55	**9**.**98**	**12**.**40**	**20**.**37**
40	15	0.26	0.92	2.27	4.93	**9**.**67**	**11**.**88**	**20**.**97**

Abbreviation: PCOS, polycystic ovary syndrome.

AMH values above the proposed cutoff in the study by Bell et al, 2021 (22) are reported in bold.

**Table 4.** Age-specific percentiles of anti-müllerian hormone in patients with PCOS for the Elecsys (Roche) assay

Age (y)	N	5th percentile	10th percentile	25th percentile	50th percentile	75th percentile	90th percentile	95th percentile
20	19	2.01	2.53	**4**.**21**	**5**.**27**	**7**.**95**	**9**.**35**	**12**.**07**
21	48	1.95	2.56	**4**.**10**	**5**.**52**	**8**.**60**	**10**.**70**	**13**.**42**
22	68	1.90	2.59	**4**.**01**	**5**.**75**	**9**.**17**	**11**.**89**	**14**.**64**
23	93	1.90	2.64	**3**.**96**	**5**.**94**	**9**.**56**	**12**.**78**	**15**.**57**
24	97	1.96	2.73	**3**.**97**	**6**.**08**	**9**.**70**	**13**.**21**	**16**.**08**
25	130	2.10	2.87	**4**.**05**	**6**.**13**	**9**.**50**	**13**.**03**	**16**.**04**
26	142	2.29	3.02	**4**.**18**	**6**.**11**	**9**.**02**	**12**.**36**	**15**.**50**
27	122	2.41	3.07	**4**.**25**	**6**.**02**	**8**.**62**	**11**.**86**	**14**.**93**
28	146	2.34	2.92	**4**.**15**	**5**.**90**	**8**.**64**	**12**.**17**	**14**.**80**
29	169	2.15	2.66	**3**.**95**	**5**.**77**	**8**.**95**	**12**.**98**	**14**.**98**
30	132	2.00	2.49	**3**.**80**	**5**.**67**	**9**.**10**	**11**.**99**	**14**.**99**
31	126	1.99	2.50	**3**.**77**	**5**.**60**	**8**.**89**	**13**.**02**	**14**.**61**
32	114	2.02	2.58	**3**.**78**	**5**.**52**	**8**.**48**	**12**.**22**	**14**.**03**
33	98	2.00	2.61	3.73	**5**.**41**	**8**.**04**	**11**.**40**	**13**.**50**
34	73	1.90	2.54	3.59	**5**.**23**	**7**.**66**	**10**.**74**	**13**.**09**
35	51	1.73	2.38	3.37	**5**.**00**	**7**.**33**	**10**.**20**	**12**.**79**
36	41	1.51	2.16	3.07	**4**.**73**	**7**.**05**	**9**.**79**	**12**.**59**
37	34	1.23	1.87	2.72	**4**.**42**	**6**.**80**	**9**.**47**	**12**.**47**
38	21	0.91	1.53	2.32	**4**.**08**	**6**.**57**	**9**.**22**	**12**.**42**
39	17	0.56	1.16	1.89	3.73	**6**.**37**	**9**.**04**	**12**.**42**
40	19	0.19	0.77	1.43	3.36	**6**.**18**	**8**.**89**	**12**.**44**

Abbreviation: PCOS, polycystic ovary syndrome.

AMH values above the proposed cutoff in the study Zhang et al, 2023 (24) are reported in bold.

**Figure 2.**
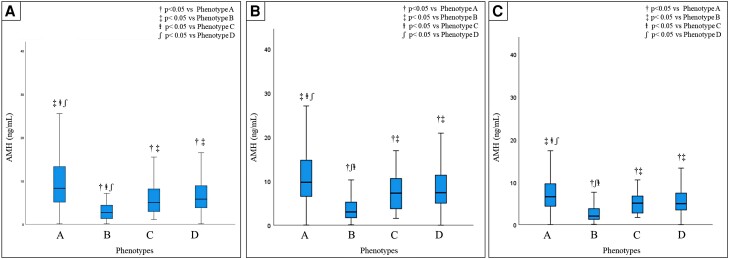
Comparison of anti-müllerian hormone levels between PCOS phenotypes for Gen II (A), picoAMH (B), and Elecsys (C) assays.

**Figure 3.**
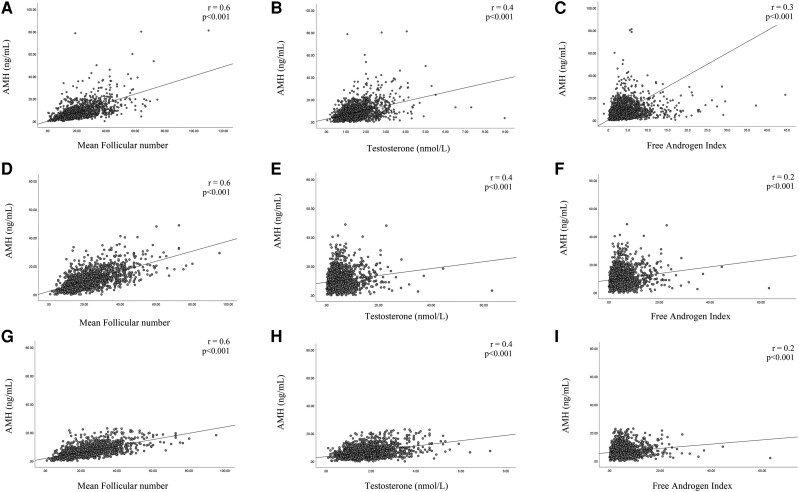
Scatterplot depicting the relationship between the individual AMH serum concentrations assessed with picoAMH (A-C), Gen II (D-F), and Elecsys assays (G-I) with mean follicle number, testosterone, and FAI, in PCOS women. Correlation coefficients and Spearman ranks (*r*) and their respective significance levels (P) are shown in the upper right corner.

The distribution of AMH (median and percentiles from the 5th to 95th) in the entire population and according to the different phenotypes for the 3 assays are summarized in Table 5. AMH levels were significantly different among PCOS phenotypes for both the assays (Fig. 2). Serum AMH levels were significantly higher in patients with phenotype A compared with all other phenotypes for all 3 assays. We also analyzed the association between serum AMH levels and BMI, endocrine, or ultrasound parameters. For all 3 assays, we found a statistically significant positive correlation between AMH levels and LH, LH/FSH ratio, testosterone, androstenedione, FAI, mean follicular number, and mean ovarian volume. In contrast, AMH levels were negatively correlated with BMI in all groups (*r* = −0.05, *P* = .03, *r* = −0.1, *P* < .001 and *r* = −0.1, *P* = .001, respectively, for Gen II, picoAMH, and Elecsys assays). The percentile distributions of mean follicular number for all groups are also reported in Fig. 1 and in Supplementary Figs. 1 and 2 (23). Figure 3 shows the main correlations between AMH and clinical, hormonal, or ultrasound parameters for the Gen II, picoAMH, and Elecsys assays.

**Table 5.** Anti-müllerian hormone distribution in the entire population and according the different phenotypes for Gen II, picoAMH, and Elecsys assays

		n	Median	5th percentile	10th percentile	25th percentile	50th percentile	75th percentile	90th percentile	95th percentile
Gen II (Beckman Coulter) assay	All patients	1702	6.9	1.9	2.6	4.3	6.9	11	17.3	21.0
	Phenotype A	962	8.3	2.4	3.3	5.1	8.3	13.3	19.6	24.3
	Phenotype B	29	2.7	0.3	0.5	1.3	2.7	4.5	6.1	8.2
	Phenotype C	103	5.0	1.5	1.9	2.9	5.0	8.3	16.2	17.9
	Phenotype D	608	5.8	1.7	2.2	3.8	5.8	8.9	12.9	15.9
picoAMH (Ansh Labs) assay	All patients	1756	8.7	2.5	3.4	5.6	8.7	13.2	18.6	21.7
	Phenotype A	973	9.7	3.1	4.4	6.5	9.7	14.7	20.3	23.9
	Phenotype B	48	3.0	0.1	1.0	1.7	3.0	5.6	8.2	11.2
	Phenotype C	44	7.3	1.7	2.2	3.7	7.3	10.6	15.7	16.7
	Phenotype D	691	7.4	2.5	3.1	5.0	7.4	11.3	16.7	19.6
Elecsys (Roche) assay	All patients	1756	5.7	1.9	5.6	3.9	5.7	8.7	12.2	14.4
	Phenotype A	973	6.6	2.5	3.1	4.2	6.6	9.6	13.2	16.4
	Phenotype B	48	2.0	0.1	0.7	1.2	2.0	4.1	5.9	7.3
	Phenotype C	44	5.0	1.8	2.0	2.7	5.0	6.7	10.1	10.4
	Phenotype D	691	4.9	1.8	2.3	3.4	4.5	7.4	10.8	13.1

## Discussion

In the past several years, AMH assessment has gained widespread use in the clinical settings of several conditions including the prediction of ovarian response in women undergoing assisted reproduction treatment, assessment of PCOS, risk of ovarian hyperstimulation syndrome, prediction of menopause, and monitoring the impact of cytotoxic chemotherapy and radiotherapy on ovarian function (25). However, the lack of an international AMH standard, even 20 years after the development of the first AMH ELISA assay, severely limits the development of AMH cutoff values that are needed for its appropriate use in clinical practice. Moreover, age-specific cutoff values are also needed and as long as the international reference is lacking, they are needed for separate assays. Previous studies evaluating age-specific AMH distribution have mainly been conducted in normo-ovulatory or infertile women, regardless of their underlying pathologies (14-17). However, to allow proper comparison of the different assays, there is an urgent need to use larger, clearly defined cohorts that are stratified by age. To our knowledge, this is the first study with real-world data reporting AMH age-specific percentile nomograms in a large population of PCOS women using three different assays (Gen II, picoAMH, and Elecsys).

In the present study, we also considered the age-percentile values of AMH in patients with PCOS for the Gen II, the picoAMH, and the Elecsys assays according to the cutoffs previously proposed in literature for each specific assay (Tables 2-4). In 2017, Lie Fong et al conducted a retrospective observational cohort study in women with PCOS (21). They proposed 2 different cutoff values for AMH evaluating through the Gen II (Beckman Coulter) assay to differentiate between patients with PCOS and regularly cycling women with normal ovaries. They distinguished young and old woman (defined as women aged ≤95th or ≥5th percentile of the corresponding cluster, respectively). In our population, we considered “young” women aged <30 years and “old” women with an age >30 years. In detail, for young women the cutoff of 5.5 μg/L presented the best specificity to discriminate PCOS patients from “normal regularly cycling non-PCOM women”, whereas for old women a cutoff of 5 μg/L was found. In Table 2, all age percentile values of AMH greater than these cutoffs are presented in bold. Interestingly, in our population, about one third of women with PCOM showed AMH levels lower than the cutoffs proposed by Lie Fong and colleagues. It is important to take into account that the vast majority of published research has evaluated which value of AMH is able to predict the diagnosis of PCOS and only a few studies have investigated the best value of AMH to predict PCOM. To our knowledge, only 1 study has examined an AMH cutoff to predict PCOM according to the picoAMH assay (22). Despite the small sample size (n = 163), Bell and colleagues reported that an Ansh assay AMH concentration ≥6.16 ng/mL (44.0 pmol/L) had a sensitive of 80.6% and a specificity of 84.8% to predict PCOM. In Table 3, we report in bold type the all AMH values above the Bell’s cutoff. Considering this cutoff, in our cohort, about one third of patients with PCOM was not correctly identified with AMH because their AMH levels were lower than cutoff proposed. Last, for the Elecsys assay, different studies have been proposed an AMH cutoff to diagnose PCOM (3, 24). The cutoff of 3.2 ng/mL was previously validated in a large cohort of women to identify PCOM (3). In detail, that cutoff had a sensitivity of 88.5% and 77.8%, and a specificity of 80.3% and 90.1% in women aged 25 to 35 and 36 to 45 years respectively, for PCOM diagnosis (3). In 2023, Zhang and colleagues published a larger retrospective study conducted on a very large cohort of women with PCOS, PCOM only, and controls to identify an Elecsys AMH cutoff value to diagnose both PCOS and PCOM. Receiver operating characteristic analysis showed that the optimal AMH threshold was 4.405 ng/mL for PCOS and 3.735 ng/mL for PCOM (24). Therefore, PCOM had a lower AMH diagnosis threshold than that for PCOS and the cutoff identified for PCOM was similar to that previously reported in the study by De Loos et al, 2021. In Table 4, AMH values lower than 3.735 ng/mL were reported in roman, whereas AMH values greater than the threshold proposed by Zhang et al are reported in bold type. Also, in this case, it is interesting to observe that using the cutoff proposed by Zhang et al, approximately one fifth with PCOM had AMH levels lower than the cutoff proposed. It is also important to underline that the reference ranges provided by manufacturers of AMH assays cannot be used as cutoffs for PCOS. They all used different cohorts, also including “healthy women” with PCOM. Additionally, it remains unclear whether women with PCOS have been excluded from these cohorts. When considering the 97.5th percentile indicated by each specific assay manufacturer, 84.4%, 74.5%, and 73.3% of women with PCOS in our cohort showed AMH levels lower than such limits for the Gen II, picoAMH, and Elecsys assays, respectively.

In addition, we found a statistically significant positive correlation between AMH serum levels and the follicle number per ovary (FNPO) and ovarian volume. Our findings are in line with a great number of previous studies that suggested a very close relationship between AMH and PCOM (3, 9, 26, 27). Figure 1 and Supplementary Figs. 1 and 2 (23) also showed the distribution of age-specific percentiles curves of FNPO in patients with PCOS. Despite some studies having also reported a discrepancy between AMH and antral follicle count (28), it was previously shown that AMH and FNPO are strictly correlated and that AMH could be even better than FNPO in diagnosing PCOM in patients with PCOS (29, 30). Accordingly, AMH has been proposed as a substitute for PCOM (26, 27), especially in situations where ultrasound is not available or not feasible. Recently, the “Recommendations From the 2023 International Evidence-based Guideline for the Assessment and Management of Polycystic Ovary Syndrome” also recognized its value, reporting that serum AMH could be used for defining PCOM in adults (31). A recent meta-analysis, including 41 studies and 13 509 women, demonstrated that replacement of PCOM in the Rotterdam criteria by serum AMH level can also predict the presence of PCOS, with a sensitivity of 78% (95% CI, 0.74-0.81), specificity of 87% (95% CI, 0.84-0.90), and an area under the receiver operating characteristic curve of 0.89 (95% CI, 0.86-0.92) (32). However, at present, there is a considerable variability between different studies assessing the role of a single AMH measurement to establish the diagnosis of PCOS (18, 31). There was also serious concern of bias in the meta-analysis conducted by Anand et al (32), thus also the recent International Evidence-based Guideline did not choose to promote the use of a single AMH measurement to diagnose PCOS in daily clinical practice (30).

In a previous study, we compared the 3 different AMH assays (Gen II-Beckman Coulter, picoAMH-Ansh Labs, and Elecsys-Roche) included in the present study, showing that the inter-assay correlation in women with PCOS was stronger in the low and high range serum AMH level subgroups (13). This means that for nearly 50% of the women included in this study, serum AMH levels could not be properly converted to an AMH value measured by other AMH assays. Furthermore, lower AMH values were measured by the Elecsys AMH assay than both the Gen II AMH and the picoAMH assays. These findings further emphasize the need of standardization of AMH measurements (13). However, despite the differences previously reported in these 3 assays (13), it is interesting to note that taking into account specific cutoffs for assay, the distribution of age percentiles values of AMH, below and above the specific cutoffs previously proposed to define PCOM, is similar for all the 3 assays analyzed (see bold AMH values in Tables 2-4). These findings allow to speculate that the distribution of PCOM in patients with PCOS in AMH age-specific percentile curves is independent from the assay used if a specific cutoff for assay is used. Indeed, all 3 assays seem to virtually distribute “PCOM” in a similar way.

As already mentioned, the cause of the raised AMH production in women with PCOS is not totally elucidated. Indeed, it appears to be not just a consequence of the increased number of AMH-producing preantral and small antral follicles (33), but also related to an intrinsic property of granulosa cells to produce higher amounts of AMH (34). Increased concentrations of AMH may also be related to other factors, including androgen production. According to previous studies (26, 35), we demonstrated a significant positive correlation between serum AMH and androgens levels. It was previously reported that women with hyperandrogenism and PCOM had higher serum concentrations of AMH than women with PCOM and normal androgen levels (36). On this basis, it might be interesting to investigate AMH levels in PCOS patients with phenotype D, which do not present with hyperandrogenism. However, only a few studies investigated AMH levels across the different PCOS phenotypes, and the results are not univocal. Piouka and colleagues reported a progressive decrease in AMH levels ranging from phenotype A to D (36). In the study conducted by Sahmay et al, the highest AMH levels were found in patients with phenotype A and lowest in patients with phenotype B (37). However, the authors did not report any statistically significant difference among phenotypes C and D. A similar AMH profile among PCOS phenotypes was also described in the study conducted by Romualdi et al (38). In accordance with previous studies, we detected the highest levels of AMH in patients with phenotype A. We have also examined in detail the cohort of patients with AMH ≤5th percentile (n = 87 with Gen II-Beckman Coulter assay, n = 84 with picoAMH-Ansh Labs, and n = 88 with Elecsys-Roche) and we found that this was not attributable to age or BMI. Interestingly, a high percentage of these patients belonged to phenotype D. These patients would not have been diagnosed with PCOS if AMH had been used instead of ultrasound.

The present study has some limitations: the cross-sectional design and the lack of individuals without PCOS and without PCOM, inclusion of which would permit characterization of the testing performance of each assay as a surrogate for PCOM. The strengths of the present study include the high number of women, well-characterized PCOS cohort, and homogenous assays for the assessment of AMH. Furthermore, patients using hormonal treatment in the past 3 months were excluded. Indeed, AMH levels are approximately 25% lower in women using hormonal contraceptives compared with nonusers (39). Longitudinal studies with a large cohort of women that could be followed for many years are needed to validate our nomogram.

## Conclusions

The appropriate identification of women with PCOS is really important in clinical practice, not only for the high prevalence of this disease, but especially because of long-term health risks. Indeed, despite the misnomer term that defines this disease, PCOS is not a problem of ovarian cysts, but a complex disorder involving reproductive, obstetric, metabolic, and cardiovascular comorbidities, which can impact women health at all ages. However, recent guidelines underlined that diagnosis of PCOS is often delayed (31). AMH testing, as a marker of PCOM, might reduce the number of women with PCOS who undergo delayed diagnosis or are undiagnosed. Indeed, several concerns still exist in detecting PCOM with ultrasound. Ultrasound is performed with different protocols all over the world. With advances in ultrasound and resolution, significant increase in FNPO has been reported with transducer frequency of >8 MHz. Thus, there are differences in the method of counting the follicles, as in the cutoff values. Furthermore, vaginal ultrasound cannot be performed in adolescents who lack sexual experience. AMH could represent a good substitute to overcome these issues. However, threshold levels can be influenced by several variables, making it difficult to identify a general cutoff.

Further research should overcome the concept of a single cutoff value. In this context, for the first time, we developed age-related tables and curves in a large cohort of PCOS patients using 3 different assays (Gen II, picoAMH, and Elecsys). Clinicians can compare an AMH value with that of a large cohort of patients with PCOS who underwent thorough phenotyping, taking account of “age” and “assay used,” two of the most important factors influencing AMH values. The results of the present study may help clinicians interpret AMH levels in patients with PCOS across age ranges.

## Data Availability

Restrictions apply to the availability of some, or all data generated or analyzed during this study to preserve patient confidentiality or because they were used under license. The corresponding author will on request detail the restrictions and any conditions under which access to some data may be provided.

## Abbreviations

AMH: anti-müllerian hormone
BMI: body mass index
FAI: free androgen index
FNPO: follicle number per ovary
OD: ovulatory dysfunction
PCOM: polycystic ovary morphology
PCOS: polycystic ovary syndrome
